# Whole-Metagenome-Sequencing-Based Community Profiles of *Vitis vinifera* L. cv. Corvina Berries Withered in Two Post-harvest Conditions

**DOI:** 10.3389/fmicb.2016.00937

**Published:** 2016-06-23

**Authors:** Elisa Salvetti, Stefano Campanaro, Ilenia Campedelli, Fabio Fracchetti, Alex Gobbi, Giovanni Battista Tornielli, Sandra Torriani, Giovanna E. Felis

**Affiliations:** ^1^Department of Biotechnology, University of VeronaVerona, Italy; ^2^Department of Biology, University of PadovaPadua, Italy; ^3^Microbion Srl, University of VeronaVerona, Italy

**Keywords:** withered grape, post-harvest, microbiome, microbial diversity, yeasts, molds, bacteria, metagenomics

## Abstract

*Vitis vinifera* L. cv. Corvina grape forms the basis for the production of unique wines, such as Amarone, whose distinctive sensory features are strongly linked to the post-harvest grape withering process. Indeed, this process increases sugar concentration and changes must characteristics. While microorganisms involved in must fermentation have been widely investigated, few data are available on the microbiota of withered grapes. Thus, in this paper, a whole metagenome sequencing (WMS) approach was used to analyse the microbial consortium associated with Corvina berries at the end of the withering process performed in two different conditions (“traditional withering,” TW or “accelerated withering,” AW), and to unveil whether changes of drying parameters could have an impact on microbial diversity. Samples of healthy undamaged berries were collected and washed, to recover microorganisms from the surface and avoid contamination with grapevine genetic material. Isolated DNA was sequenced and the data obtained were analyzed with several bioinformatics methods. The eukaryotic community was mainly composed by members of the phylum Ascomycota, including Eurotiomycetes, Sordariomycetes, and Dothideomycetes. Moreover, the distribution of the genera *Aspergillus* and *Penicillium* (class *Eurotiomycetes*) varied between the withered berry samples. Instead, *Botryotinia, Saccharomyces*, and other wine technologically useful microorganisms were relatively scarce in both samples. For prokaryotes, 25 phyla were identified, nine of which were common to both conditions. Environmental bacteria belonging to the class Gammaproteobacteria were dominant and, in particular, the TW sample was characterized by members of the family Pseudomonadaceae, while members of the family Enterobacteriaceae dominated the AW sample, in addition to Sphyngobacteria and Clostridia. Finally, the binning procedure discovered 15 putative genomes which dominated the microbial community of the two samples, and included representatives of genera *Erwinia, Pantoea, Pseudomonas, Clostridium, Paenibacillus*, and of orders Lactobacillales and Actinomycetales. These results provide insights into the microbial consortium of Corvina withered berries and reveal relevant variations attributable to post-harvest withering conditions, underling how WMS could open novel perspectives in the knowledge and management of the withering process of Corvina, with an impact on the winemaking of important Italian wines.

## Introduction

Grape naturally hosts a reservoir of microorganisms that may be transferred into the winery and affect the vinification process, influencing wine quality and storage (Mills et al., 2007). The microbial population of sound grape berry is roughly comprised between 10^3^ and 10^5^ cfu/g (Barata et al., 2012), and includes filamentous fungi, yeasts, and bacteria with different physiological characteristics and different impact on the grape metabolome and final wine quality (Verginer et al., 2010; Pinto et al., 2015). Some species are found only in grapes, as saprophytic molds, like *Aspergillus* spp., *Cladosporium* spp., and *Penicillium* spp. (Martins et al., 2014), and environmental bacteria, while others are able to survive and grow in wine, constituting the wine microbial consortium (Barata et al., 2011; Liu et al., 2015), that comprises yeasts, lactic acid bacteria (LAB) and acetic acid bacteria (AAB).

Yeast species present on the berry may play important roles during the alcoholic fermentation and have significant impact on quality and aromatic properties of wine (Pretorius, 2000; Fleet et al., 2002). Species present on sound ripe berries have been reported to belong mainly to the group of oxidative basidiomycetous yeasts, such as *Cryptococcus* spp., *Rhodotorula* spp., *Sporobolomyces* spp., and *Filobasidium* spp., as well as to the dimorphic ascomycetous black yeast, *Aureobasidium pullulans* (Prakitchaiwattana et al., 2004; Magyar and Bene, 2006; Barata et al., 2008). These yeasts are ubiquitous in the vineyard environment and they are typically associated with grapes, phyllosphere, and soil (Setati et al., 2012; Gilbert et al., 2014). The oxidative ascomycetous yeasts (e.g., *Candida* spp., *Pichia* spp., and *Metschnikowia* spp.), and the fermentative ascomycetous yeasts (e.g., *Hanseniaspora*/*Kloeckera* spp.) have been found to be present at low concentrations on sound berries and appear often localized in those areas of the grape surface where some juice might escape (Nisiotou and Nychas, 2007; Čadež et al., 2010; Capozzi et al., 2015). In contrast, *Saccharomyces cerevisiae*, the most relevant fermentative wine yeast, is mostly present in low number and low frequencies, even in damaged berries (Fleet, 2003).

Grapes are considered the primary source of LAB (Nisiotou et al., 2011), which catalyse the conversion of L-malic acid to L-lactic acid with the production of CO_2_ through malolactic fermentation and to impart flavor complexity (Sumby et al., 2014). Species belonging to the LAB group, such as *Lactobacillus* spp., *Leuconostoc* spp., *Pediococcus* spp., and *Oenococcus oeni*, have been frequently found in wine and winery (Pérez-Martín et al., 2014; Sumby et al., 2014), but they have been isolated from sound or damaged berries only rarely (Barata et al., 2012). Grapevine microbiota also shows a broad diversity of ubiquitous environmental bacteria belonging to the genera *Bacillus, Burkholderia, Enterobacter, Enterococcus, Pseudomonas, Serratia*, and *Staphylococcus* (Leveau and Tech, 2011; Martins et al., 2012; Gilbert et al., 2014), which are unable to grow in wine. In addition to LAB and environmental bacteria, the genera *Acetobacter, Gluconoacetobacter*, and *Gluconobacter* belonging to the AAB group have also been found on the grape surfaces (Barbe et al., 2001; Barata et al., 2012). AAB are well known for their ability to produce acetic acid from sugars and through the oxidation of the ethanol, representing a key factor in wine spoilage (Bartowsky and Henschke, 2008).

The microbiota of grapes is highly variable, mostly due to the influence of external factors, as environmental parameters, geographical location, and grape cultivars (Bokulich et al., 2013, 2014; Pinto et al., 2014). *Vitis vinifera* L. cv. Corvina is the most important red grape variety of the Verona area in north Italy, displaying good vigor, providing abundant, fairly consistent yields, and showing fair resistance to disease and hardship (Andreolli et al., 2016). The fundamental role of this grapevine variety in conferring the uniqueness wine aroma has been underlined by previous transcriptomic, proteomic, and metabolomic studies (Di Carli et al., 2011; Toffali et al., 2011; Dal Santo et al., 2013; Venturini et al., 2013). This late-ripening variety forms the basis of Verona's red wines and, despite showing a certain fragility during the drying process, it is essential in the production of Amarone wine, to which it gives structure, weight, and a surprising softness (Paronetto and Dellaglio, 2011). The distinctive features of such wine are strongly linked to the post-harvest withering process, an ancient local technique of grape semi-drying, which goes as far back as the Romans time. The grapes are partially dried in large, well-aired rooms (*fruttaio*) for 2/3 months, causing elimination of water, concentration of sugar up to about 30% (w/v) and other substances, evolution of aromatic molecules and phenolic compounds (Consonni et al., 2011; Tosi et al., 2012). Therefore, the drying process leads to a large number of changes in the grape and must characteristics, depending on environmental parameters (temperature, humidity, ventilation), time, and microbial activities. However, few studies have been carried out on the microbial communities associated with withered berries (Lorenzini et al., 2013; Rantsiou et al., 2013; Guzzon et al., 2014), and the effects of withering conditions on overall grape microbiota is still largely unknown.

The development of next-generation sequencing provides a useful tool for the description of prokaryotic and eukaryotic microbial communities associated with grape berries, leaves, must and wineries (Bokulich et al., 2013, 2014; David et al., 2014; Pinto et al., 2014, 2015; Taylor et al., 2014; Piao et al., 2015; Wang et al., 2015). Microbial community profiling using whole metagenome sequencing (WMS) could allow an accurate and detailed investigation of the underlying microbial community, providing data also for minority species (Thomas et al., 2012). Contrary to rDNA-targeting pyrosequencing (including both 16S and 18S rRNA genes, as well as the nuclear ribosomal internal transcribed spacer regions), metagenomics offers the possibility to describe diversity at genome level, also revealing the functional gene composition of microbial communities (Sharpton, 2014). Moreover, genomic information acquired from metagenomic sampling can contribute substantially in the recognition of new taxa (Ladoukakis et al., 2014) and improve the *Candidatus* proposal, a provisional status for uncultivated novel taxa (Konstantinidis and Rosselló-Móra, 2015).

In this study, a WMS approach was carried out with the primary aim of analyzing the diversity of the microbial consortia of Corvina sound berry surfaces at the end of the withering process. Only healthy berries were considered to get information on their “natural” microbiota that might play an important role in the metabolic processes taking place inside the berries during withering. Two different post-harvest withering conditions were analyzed (“traditional,” TW, and “accelerated,” AW) to unveil whether changes of drying parameters could lead to relevant modifications of the microbial components in this peculiar ecological niche.

## Materials and methods

### Grape withering and sampling

The collection of grapevine (*V. vinifera* L. cv. Corvina) bunches was carried out at the *fruttaio* located in Gargagnago di Sant'Ambrogio di Valpolicella, Italy (45°31′20″ N, 10°50′05″ E). Grapes, harvested during the 2013 vintage, were placed on wooden racks in the *fruttaio* and subjected to two different withering conditions, i.e., “TW” and “AW.” Temperature, relative humidity, and ventilation were set up to maintain conventional parameters, i.e., a gradually decreasing temperature (from 16 to 8°C) and a gradually increasing relative humidity (from 60 to 80%; http://www.appaxximento.it/eng/#fruttai) for the TW berry batch; while, a fan was placed close to another batch of grapes to promote a faster drying (AW condition). In this way, AW grapes were exposed to an average airflow of about 1 m/s that in turn contributed to remove part of the humid air stacking around the clusters, almost without affecting temperature. Temperature and relative humidity close to AW grapes were on average 0.2 ± 0.5°C and 13.5 ± 8.5% lower than TW grapes, respectively.

Grape weight loss was monitored during the withering process, and bunches were randomly sampled when grapes reached ~30% of the weight loss (i.e., after 61 and 109 days for AW and TW conditions, respectively) and they were ready for the crushing stage. Only healthy undamaged bunches were used for the analysis. Grape bunches were placed in sterile plastic bags and transferred to the laboratory in a refrigerated container. Under aseptic conditions in the laboratory, sound berries were harvested, gentle destemming, separating stems from berries, pooled together (150 g) in order to make the sample representative, and processed as described below.

### Microbial cell collection, genomic DNA extraction, and sequencing

Berries were processed according to Renouf et al. (2005) with slight modifications. Basically, berries were placed in a sterile 500 mL flask containing a solution of Bacto Soytone (Sigma-Aldrich, St. Louis, MO, USA; 10 g/L) and Tween 80 (Sigma-Aldrich; 2 mL/L) to wash them and to release the microorganisms from the surface. This step was carried out twice at 20°C for 3 h with slow shaking. The washing solutions were then filtered through 0.45 μm Whatman nitrocellulose membrane filters (Sigma-Aldrich) and stored at 4°C until DNA extraction.

Total genomic DNA was extracted from the two filter membranes independently using the PowerWater® DNA Isolation Kit (MO BIO Laboratories Inc., Carlsbad, CA, USA), according to the manufacturer's instructions. The quantification and quality control of the DNA was determined with a 2100 Bioanalyzer (Agilent Technologies, Santa Clara, CA, USA). The concentration of the DNA samples was normalized and the sequencing was carried out at the Functional Genomics Centre (University of Verona, Verona, Italy) using an Illumina HiSeq 2000 (Illumina Inc., San Diego, CA, USA) platform which generated 2 × 100-bp pair-end sequencing reads.

### Bioinformatics analysis

#### Reads trimming and de novo metagenome assembly

Reads in FASTQ format were trimmed using Trimmomatic software version 0.35 (Bolger et al., 2014; considering quality encoding phred 33) with the following parameters: LEADING:3 TRAILING:3 SLIDINGWINDOW:4:15 MINLEN:30 SLIDINGWINDOW:4:15 MINLEN:30.

The presence of contaminant sequences derived from *V. vinifera* was determined aligning the filtered reads with Bowtie2 software version 2.2.6 (Langmead and Salzberg, 2012) on the reference grape genome (Jaillon et al., 2007) downloaded from NCBI database. Unaligned reads were extracted from the bam file using bedtools software package v2.17.0 (Quinlan and Hall, 2010).

Reads were assembled with MetaVelvet software version 1.2.02 (Namiki et al., 2012), using a kmer of 63 and a minimum scaffold length of 500 bp. When both paired sequences passed the trimming and quality check, they were used as paired-end (with insert size equal to 445 and SD 140), while sequences where only one pair passed the filtering step were used in the assembly as shotgun. Assembly was performed using the option “-exp_cov auto” in order to perform the final assembly step with MetaVelvet. MetaVelvet assembly was performed using the parameter “-exp_covs” and considering coverage peaks 1100, 615, 273, 110, 41, 24, and 7.5. Scaffolds were filtered and renamed using the perl script “rename_fasta_file.pl” (Campanaro et al., 2016).

#### Gene finding and annotation

Gene finding on the scaffolds obtained from the assembly process was performed running the program Prodigal in metagenomic mode (Hyatt et al., 2012). Conserved protein families and domains were identified using reverse position-specific BLAST algorithm (RPSBLAST; NCBI BLAST+) on all the proteins predicted from assembly and using COG-only (Galperin et al., 2015), Pfam (Finn et al., 2016), CDD (Marchler-Bauer et al., 2015), eggNOG (Powell et al., 2013), rpsBLAST databases. Only results with *e*-value lower than 1e^−5^ were considered; for COG, CDD and eggNOG only the best match was considered. KEGG annotation was performed using KEGG Automatic Annotation Server (KAAS; Moriya et al., 2007). After the binning process, scaffolds assigned to each genome bin were re-annotated using the Rapid Annotation Subsystem Technology (RAST) server (Overbeek et al., 2014). We further assigned a rough gene descriptions using BLASTp analysis performed on a database containing all the sequences of 2,765 complete bacterial genomes downloaded from NCBI database, only results with *e*-value lower than 1e^−5^ were considered.

#### Calculation of the scaffold coverage

Reads obtained individually for the two samples analyzed were aligned to the scaffolds larger than 500 bp with the Bowtie2 software version 2.2.6 and coverage was determined with the genomecov software of the bedtools package (Quinlan and Hall, 2010), “calculate_coverage_fromsam.pl” and “average_coverage_bedtools.pl” perl scripts (Campanaro et al., 2016). Coverage was normalized considering the number of aligned reads and using the sample with the lower number as a reference. The coverage obtained was considered for comparison between number of genes for each KEGG pathway and their average coverage.

#### Binning of genomes using tetranucleotide composition and coverage

Binning process was performed using the procedure of Albertsen et al. (2013) which is based on sequence composition-independent binning and tetranucleotide binning. In the first step distinct groups of scaffolds were identified for their coverage similarity in the two samples (AW and TW). Bin selection was facilitated by coloring scaffolds according to their taxonomic affiliation. In the second step, principal component analysis (PCA) of tetranucleotide frequencies was used to separate species present in the same coverage-defined bin. Scaffolds missed in the binning process were recovered and the paired-end connections between scaffolds were checked using the script “cytoscapeviz.pl” (Albertsen et al., 2013) and “recover_interacting_scaffold.pl”.

#### Identification of conserved marker genes

A set of HMMs of essential single-copy genes (Dupont et al., 2012) were searched against the predicted open reading frames (ORFs) using HMMER3 (http://hmmer.janelia.org/) with the strategy proposed by Albertsen et al. (2013). The number of the essential genes in the genomic bins identified allowed the prediction of genome completeness and the duplication level using the script “extract_data_from_contigs_list.pl”. For a proper calculation of the completeness, 105 essential genes of Firmicutes, Gammaproteobacteria, and Actinobacteria taxonomic groups were considered. The coverage of the genome bins was determined using the script “calculate_genome_coverage.pl”.

#### Taxonomic annotation for the genome bins

Taxonomic analysis of the bacterial genome bins was examined by different methods and the results were compared to obtain a consensus assignment. In this analysis, only prokaryotic species were examined because the low abundance of the eukaryotic species prevented their assembly and binning. The essential genes associated to each genome bin were checked by sequence similarity to the non-redundant (*nr*) database using BLASTn, with *e*-value threshold equal to 1e^−5^. Average sequence similarity of 95, 85, and 75% or better on the essential genes was used for species, genus and phylum level taxonomic annotation, respectively (Nielsen et al., 2014). The results, obtained from BLASTp performed on all the proteins predicted on the database of the complete microbial proteomes, were also checked to obtain information on the most similar species. Moreover, proteins encoded by genome bins were fed into Phylophlan version 0.99 to accurately determine their taxonomic identities (Segata et al., 2013). This software identifies hundreds of conserved proteins from a catalog of more than 3,700 finished and draft microbial genomes and uses them to build a high-resolution phylogeny. Results obtained were separated in “high,” “medium,” “low,” and “incomplete” confidence. The high-resolution microbial tree of life with taxonomic annotations was obtained using standard parameters.

#### Metagenomic analysis of the shotgun reads

Shotgun reads were used to profile the composition of the microbial community using MetaPhlAn version 1.7.7 (Segata et al., 2012). The software was run with standard parameters but using—sensitive-local in the Bowtie2 alignment step.

Moreover, two millions of the reads not aligned to the assembly were uploaded on the Metagenome MG Rapid Annotation using Subsystem Technology (MG-RAST) database server (http://metagenomics.anl.gov/; Meyer et al., 2008) and they were dereplicated (Gomez-Alvarez et al., 2009). The sequences which aligned to *Homo sapiens* NCBI v36 genome were removed (Langmead et al., 2009) together with the low quality sequences identified using a modified dynamic trim (Cox et al., 2010). The number of sequences obtained for each taxonomic group was determined with the MG-RAST toolkit using default parameters and selecting “RefSeq” and “GenBank” as annotation sources.

#### Nucleotide sequence accession numbers

Shotgun reads were assigned to the study PRJNA289617 with ID number SRP063004 and were deposited to NCBI Sequence Reads Archive (SRA) with the following accession numbers: traditional withering (sample SRS1050145; experiment SRX1175002; run SRR2219805) and accelerated withering (sample SRS1050146; experiment SRX1175003; run SRR2219866). Metagenome assembly was deposited at DDBJ/ENA/GenBank under the accession LIDZ00000000. The version described in this paper is version LIDZ01000000. The two million reads that are not represented in the metagenome assembly were deposited to MG-RAST database and are freely accessible with the following ID numbers: 4580981.3 and 4580980.3 for the TW and AW samples, respectively.

Genome bins extracted from the assembly with the binning process are available on the RAST database (http://rast.nmpdr.org/rast.cgi?page=Home).

## Results

### Metagenomic sequencing and analysis of the berry microbiota

A total of 89,280,038 and 62,412,964 sequences were generated from the grape cv. Corvina subjected to the two different post-harvest withering conditions, AW and TW, respectively. Only 223,038 (0.25%) and 163,898 (0.26%) sequences were removed from AW and TW-derived data on the basis of quality control parameters, highlighting the success of the sequencing. Moreover, only the 0.066% (58,555 reads of AW data) and the 0.018% (11,024 reads of TW data) of the sequences showed similarity to grapevine sequences, as detected by Bowtie2-derived alignments against the reference genome of *V. vinifera* (*V. vinifera* 12X; Jaillon et al., 2007), demonstrating the very low level of plant DNA contamination due to the washing procedure used for the isolation of the berry microbiota.

Several analyses were performed to analyse the characteristics of the berry microbial communities: (i) mapping shotgun reads against MG-RAST database and a database of species-specific genomic regions; and (ii) assembling sequences into scaffolds. Since short reads could be error-prone and could contain low signal for homology search, the generation of longer sequences can simplify bioinformatics analyses. The assembly of the reads was performed with MetaVelvet including all the sequences (pair-end, and those where only forward or reverse pairs remained after filtering). A large fraction of the reads (~96%) was assembled in scaffolds ≥500 bp (15,893 scaffolds including 86,379,522 bp; Figure S1).

A preliminary analysis of the assembly revealed that the berry microbiota was dominated by individual draft genomes belonging to prokaryotic communities. Therefore, the taxonomic diversity of the prokaryotic fraction was characterized through the analysis of the shotgun reads and the near-complete draft genomes of the species that dominated the samples obtained from the assembly. Since the scaffolds were not assigned to the eukaryotic fraction, we inferred that the fungal microbiome were not assembled due to their low abundance within the two samples, thus representing the “rare biosphere.” Therefore, the taxonomic profiling of the eukaryotic population was typified examining only the reads that were not included in the assembly.

Although the primary aim of the present paper was the analysis of the microbial biodiversity, a preliminary investigation of the functional properties of the biotic consortia was carried out: genes were predicted using the program Prodigal, and ORFs were annotated through BLASTp analyses against a database composed by the protein sequences encoded by 2,765 prokaryotic genome sequences available in NCBI. In addition, the reverse position-specific BLAST algorithm (RPSBLAST) was used on all predicted proteins using COG-only and Pfam rpsBLAST databases.

The 86,425 protein encoding genes were annotated using COG, KEGG, eggNOG and Pfam; 57,333 genes (66.3%) had a match in the COG database; 23,220 (26.9%) in KEGG, and 59,401 (68.7%) had a protein domain annotated in Pfam; 63,154 had a match in the eggNOG database (73.1%), and 76,407 (88.4%) found a match in the BLASTp comparisons against the proteins encoded in the complete microbial genomes of the NCBI database (Table S1).

Analysis of the number of genes belonging to each COG and KEGG categories in the assembly was compared with the abundance (i.e., the number of gene copies) calculated considering coverage obtained for each scaffold in the two withering conditions examined. This allowed an evaluation of the relevance of the COG/KEGG classes considering the number of genes in the pathways and their abundance in the species. From these data, it is evident that some COG categories and metabolic pathways include genes with a high average coverage, which are encoded in the genomes of the most abundant species.

**Figure 1:**
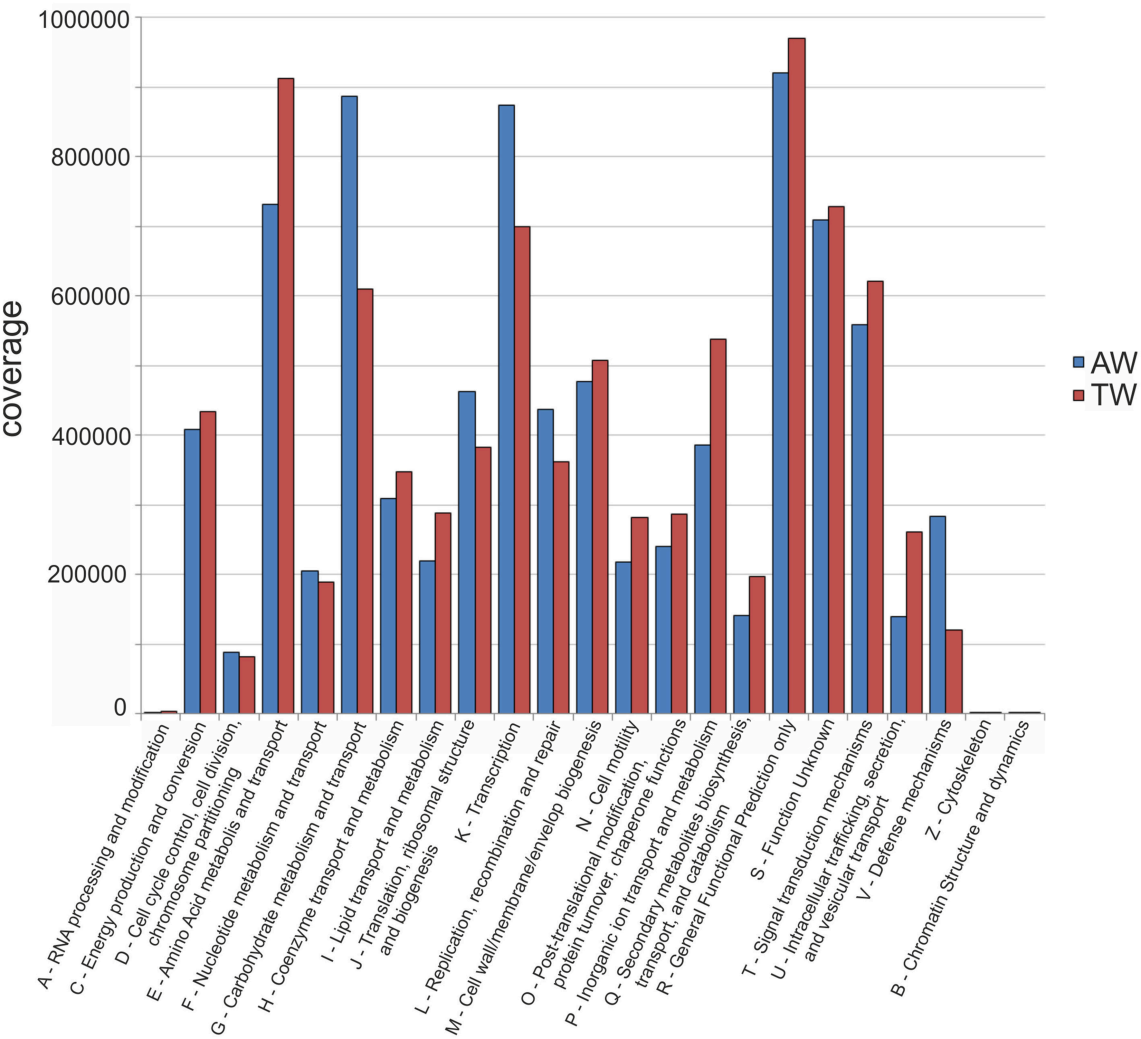
**Coverage of the functional categories found in the two metagenomic datasets**. A, RNA processing and modification; B, chromatin structure and dynamics; C, energy production and conversion; D, cell cycle control, cell division, chromosome partitioning; E, amino acid transport and metabolism; F, nucleotide transport and metabolism; G, carbohydrate transport and metabolism; H, coenzyme transport and metabolism; I, lipid transport and metabolism; J, translation, ribosomal structure, and biogenesis; K, transcription; L, replication, recombination, and repair; M, cell wall/membrane/envelope biogenesis; N, cell motility; O, post-translational modification, protein turnover, and chaperones; P, inorganic ion transport and metabolism; Q, secondary metabolites biosynthesis, transport, and catabolism; R, general function prediction only; S, function unknown; T, signal transduction mechanisms; U, intracellular trafficking, secretion, and vesicular transport; V, defense mechanisms; W, extracellular structures; Y, nuclear structure; Z, cytoskeleton.

The coverage of the functional categories found in the two metagenomic datasets is shown in Figure 1. The categories had similar abundances in both samples. For all genes clustered in COG categories, the main categories were E (aminoacid metabolism and transport), G (carbohydrate metabolism and transport), K (transcription), T (signal transduction mechanisms), R (general function prediction only), and S (function unknown).

Considering all categories, those more numerically different between the AW and TW samples were represented by E (aminoacid metabolism and transport), G (carbohydrate metabolism and transport), K (transcription), U (intracellular trafficking, secretion, and vesicular transport), and V (defense mechanisms). In particular, within the E category, elements of the histidine permease ABC transporter, such as the genes *HisJ* and *HisM*, were the most abundant in both samples. Regarding the G category, the gene *araJ*, encoding for an arabinose efflux permease, was the predominant in both samples, while genes coding for the ABC-type sugar transport system, such as *UgpA, UgpB*, and *UgpE*, were mainly present in the TW sample respect to the AW sample. It is also interesting the presence of numerous genes involved in arginine transport and metabolism, in particular *ArtQ* (18 genes having higher coverage in TW, and 10 genes in AW), *ArtM* (17 genes in TW, and 8 genes in AW), *ArgF* (11 genes in TW, and 10 genes in AW), and *ArcC* (1 gene in TW, and 7 genes in AW).

### Composition profiling of microbial communities

**Figure 2:**
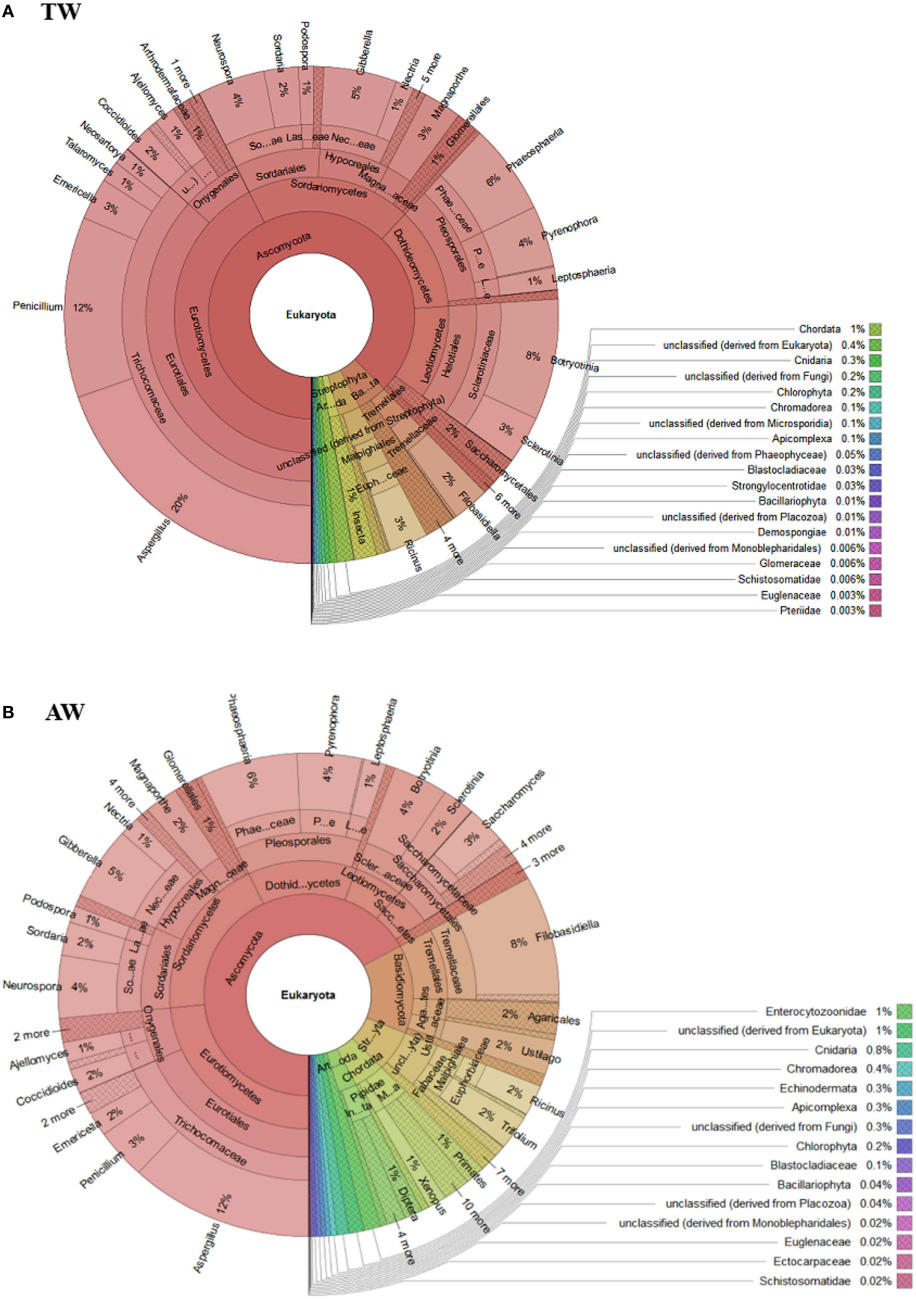
**The ecological diversity of the eukaryotic population of traditional (A-TW) and accelerated (B-AW) withered berry samples estimated using MG-RAST software based on 2 million reads selected from each samples querying the NCBI *nr* database**.

The eukaryotic population diversity was estimated using MG-RAST software based on 2 million reads selected from each sample, which were not included into the scaffolds obtained from the assembly. The unassembled reads represented the 2.9% (5,145,300 reads) for TW and 2.1% (2,598,617 reads) for AW samples. This approach revealed that the eukaryotic community was mainly composed by members of the phylum Ascomycota, 85% (33,191 reads) and 62% (3,023 reads) of reads in the TW and AW samples, respectively (Figure 2). This evidenced that, despite the strong sequencing effort, the absolute number of reads assigned to the fungal species remained low and this prevented their assembly and the functional analysis, which was limited to the prokaryotic fraction of the microbiome.

**Figure 3:**
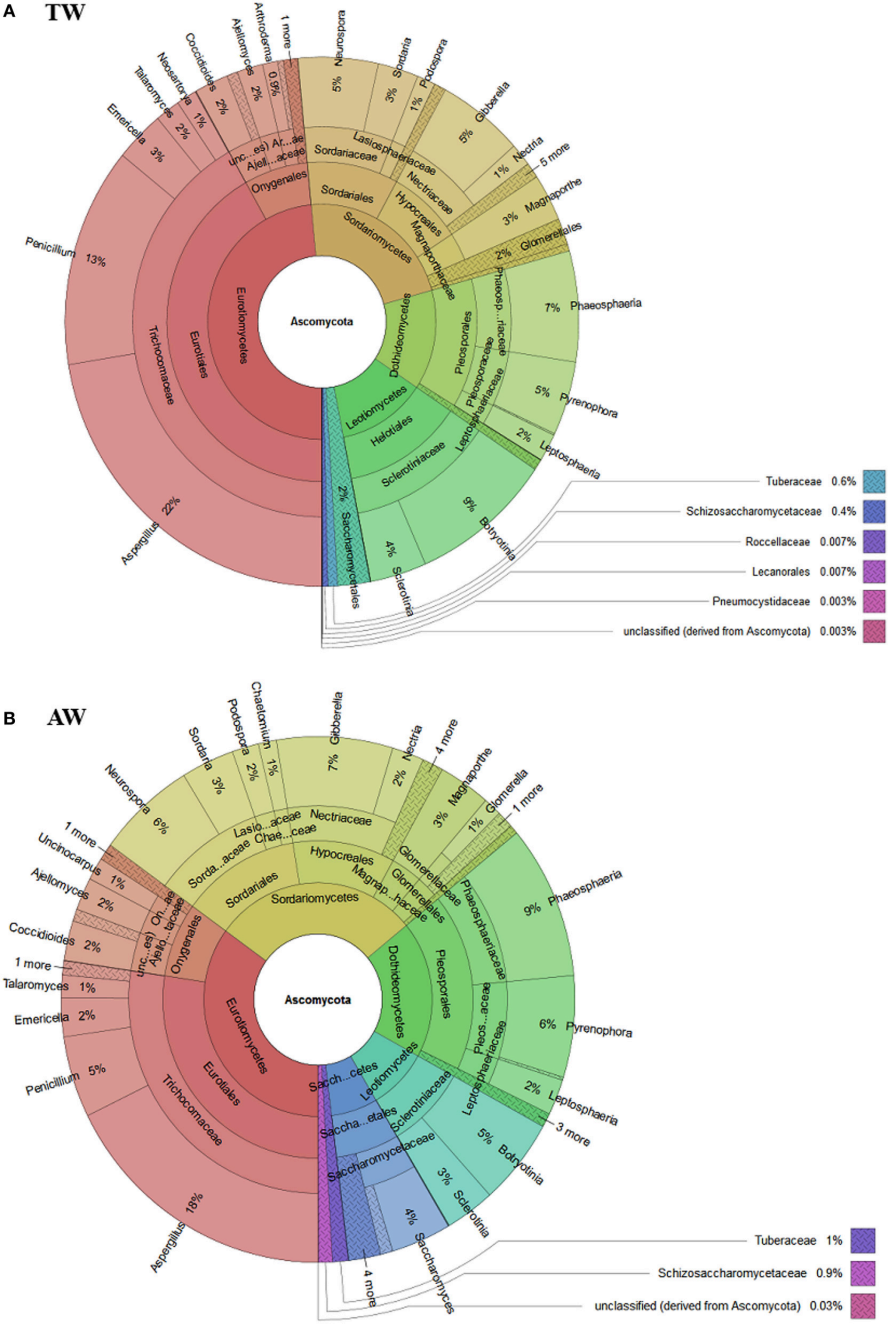
**The composition of the Ascomycota population of traditional (A-TW) and accelerated (B-AW) withered berry samples estimated using MG-RAST software based on 2 million reads selected from each samples querying the NCBI *nr* database**.

In detail, in both metagenomic datasets the majority of ascomycetes belong to the class Eurotiomycetes, in particular to the genera *Aspergillus* and *Penicillium*, but also to classes Sordariomycetes (principally the genera *Neurospora* and *Gibberella*), and Dothideomycetes (specifically the genera *Phaeosphaeria* and *Pyrenophora*; Figure 3).

The abundance of fungal population belonging to the genera *Neurospora, Gibberella* (whose anamorph is *Fusarium* sp.), *Phaeosphaeria*, and *Pyrenophora* was similar in the two samples. Conversely, the distribution of the genera of the class Eurotiomycetes (47% of the Ascomycota fraction for the TW berries; 15,436 reads), such as *Aspergillus* and *Penicillium*, varied between the two samples, representing, respectively, the 22% (6,428 reads) and 13% (4,212 reads) for the TW sample (Figure 3A-TW) and the 18% (463 reads) and 5% (161 reads) for the AW sample (Figure 3B-AW). Moreover, members belonging to the genus *Botryotinia* were detected in both samples: they constituted the 9% and 5% of the Ascomycota population of the AW and TW samples, respectively.

The bioinformatics analyses of the metadata did not reveal the presence of yeasts commonly associated with sound berries, such as *Aureobasidium, Cryptococcus, Hanseniapora, Metschnikowia*, and *Sporobolomyces*. Moreover, low amounts of wine yeast species with important role in winemaking were retrieved: members of the order Saccharomycetales represented approximately 2% (57 reads) and 1% (446 reads) of the Ascomycota fraction of the AW and TW samples, respectively. Interestingly, both samples showed the presence of members of the genus *Saccharomyces* (0.3 and 0.2% of the Ascomycota fraction of the AW and TW samples, respectively; 8 and 62 reads), which includes the most important yeasts for Amarone wine production, i.e., *S. cerevisiae* and *Saccharomyces bayanus* (Torriani et al., 1999).

The prokaryotic taxonomic diversity was characterized by aligning reads obtained from each sample against a dataset of clade-specific marker sequences, which unequivocally identified specific microbial clades at the species level or higher taxonomic ranks (Table S2; Segata et al., 2012). A total of 25 phyla were detected at the end of drying process performed in the two different conditions, of which nine phyla were found on the berry surfaces from both samples: i.e., Acidobacteria, Actinobacteria, Bacteroidetes, Chlamydiae, Chloroflexi, Cyanobacteria, Firmicutes, Proteobacteria, and Thermi (Table 1). Moreover, the relative abundance of each taxonomic unit was provided, revealing that Proteobacteria was the predominant phylum in both samples (97.7 and 86.1% for the TW and AW berries, respectively). Less abundant phyla were Firmicutes (7.8%), and Actinobacteria (0.7%) in the AW and TW samples, respectively; while Bacteroidetes was 4.7% in the AW and 1.2% in the TW conditions.

**Table 1:** **Relative abundance of prokaryotic phyla associated with grape surfaces of the traditional and accelerated withering process obtained through the MetaPhlAn analyses**.

**ID**	**Traditional withering**	**Accelerated withering**
k__Bacteria;p__Proteobacteria	97.6583	86.0501
k__Bacteria;p__Bacteroidetes	1.2162	4.7371
k__Bacteria;p__Actinobacteria	0.7383	0.0179
k__Bacteria;p__Chlamydiae	0.1423	0.4725
k__Bacteria;p__Firmicutes	0.0833	7.7888
k__Bacteria;p__Chloroflexi	0.0536	0.3574
k__Bacteria;p__Thermi	0.0431	0.0111
k__Bacteria;p__Cyanobacteria	0.0180	0.0009
k__Bacteria;p__Acidobacteria	0.0140	0.0128
k__Bacteria;p__Verrucomicrobia	0.0075	0.0000
k__Bacteria;p__Synergistetes	0.0061	0.0000
k__Bacteria;p__Gemmatimonadetes	0.0033	0.0000
k__Bacteria;p__Chlorobi	0.0021	0.0000
k__Bacteria;p__Planctomycetes	0.0021	0.0000
k__Bacteria;p__Lentisphaerae	0.0014	0.0000
k__Bacteria;p__Chrysiogenetes	0.0005	0.0000
k__Bacteria;p__Aquificae	0.0000	0.0042
k__Bacteria;p__Deferribacteres	0.0000	0.0028
k__Bacteria;p__Dictyoglomi	0.0000	0.0008
k__Bacteria;p__Fusobacteria	0.0000	0.2326
k__Bacteria;p__Nitrospirae	0.0000	0.0009
k__Bacteria;p__Spirochaetes	0.0000	0.0257
k__Bacteria;p__Tenericutes	0.0000	0.1460
k__Bacteria;p__Thermotogae	0.0000	0.0101
k__Bacteria;p__WWE1	0.0000	0.0001

At the class level, the prokaryotic communities associated with berries of the TW and AW conditions were mostly characterized by Gammaproteobacteria (94.1 and 84.9%, respectively). Moreover, minor abundance of Clostridia, Sphingobacteria, and Bacilli characterized the AW sample (6.5, 4.6, 1.3%, respectively; Table 2).

**Table 2:** **Relative abundance of prokaryotic classes associated with grape surfaces of the traditional and accelerated withering process obtained through the MetaPhlAn analyses**.

**ID**	**Traditional withering**	**Accelerated withering**
k__Bacteria;p__Proteobacteria;c__Gammaproteobacteria	94.0720	84.9150
k__Bacteria;p__Proteobacteria;c__Alphaproteobacteria	1.3884	0.1623
k__Bacteria;p__Bacteroidetes;c__Sphingobacteria	1.2101	4.6223
k__Bacteria;p__Proteobacteria;c__Deltaproteobacteria	1.1684	0.4152
k__Bacteria;p__Proteobacteria;c__Betaproteobacteria	1.0192	0.5203
k__Bacteria;p__Actinobacteria;c__Actinobacteria	0.7383	0.0179
k__Bacteria;p__Chlamydiae;c__Chlamydiae	0.1423	0.4725
k__Bacteria;p__Chloroflexi;c__Thermomicrobia	0.0465	0.3569
k__Bacteria;p__Firmicutes;c__Clostridia	0.0359	6.48421
k__Bacteria;p__Firmicutes;c__Bacilli	0.0349	1.29394
k__Bacteria;p__Thermi;c__Deinococci	0.0344	0.01114
k__Bacteria;p__Cyanobacteria;c__Cyanophyceae	0.0157	0.0009
k__Bacteria;p__Firmicutes;c__Negativicutes	0.0126	0.0070
k__Bacteria;p__Acidobacteria;c__Acidobacteria	0.0118	0.0128
k__Bacteria;p__Thermi;c__Thermi	0.0088	0.0000
k__Bacteria;p__Proteobacteria;c__Epsilonproteobacteria	0.0076	0.0369
k__Bacteria;p__Bacteroidetes;c__Bacteroidia	0.0061	0.0191
k__Bacteria;p__Synergistetes;c__Synergistia	0.0061	0.0000
k__Bacteria;p__Verrucomicrobia;c__Opitutae	0.0043	0.0000
k__Bacteria;p__Chloroflexi;c__Chloroflexi	0.0036	0.0000
k__Bacteria;p__Gemmatimonadetes;c__Gemmatimonadetes	0.0033	0.0000
k__Bacteria;p__Verrucomicrobia;c__Spartobacteria	0.0029	0.0000
k__Bacteria;p__Proteobacteria;c__Zetaproteobacteria	0.0028	0.0004
k__Bacteria;p__Chloroflexi;c__Anaerolineae	0.0024	0.0005
k__Bacteria;p__Cyanobacteria;c__Gloeobacteria	0.0023	0.0000
k__Bacteria;p__Acidobacteria;c__Solibacteres	0.0022	0.0000
k__Bacteria;p__Chlorobi;c__Chlorobia	0.0021	0.0000
k__Bacteria;p__Planctomycetes;c__Planctomycetacia	0.0021	0.0000
k__Bacteria;p__Lentisphaerae;c__Lentisphaerae_uncl	0.0014	0.0000
k__Bacteria;p__Chloroflexi;c__Dehalococcoidetes	0.0011	0.0000
k__Bacteria;p__Chrysiogenetes;c__Chrysiogenetes	0.0005	0.0000
k__Bacteria;p__Verrucomicrobia;c__Verrucomicrobiae	0.0003	0.0000
k__Bacteria;p__Aquificae;c__Aquificae	0.0000	0.0042
k__Bacteria;p__Bacteroidetes;c__Cytophagia	0.0000	0.0002
k__Bacteria;p__Deferribacteres;c__Deferribacteres	0.0000	0.0028
k__Bacteria;p__Dictyoglomi;c__Dictyoglomia	0.0000	0.0008
k__Bacteria;p__Firmicutes;c__Erysipelotrichi	0.0000	0.0037
k__Bacteria;p__Bacteroidetes;c__Flavobacteria	0.0000	0.0956
k__Bacteria;p__Fusobacteria;c__Fusobacteria	0.0000	0.2326
k__Bacteria;p__Tenericutes;c__Mollicutes	0.0000	0.1460
k__Bacteria;p__Nitrospirae;c__Nitrospira	0.0000	0.0009
k__Bacteria;p__Spirochaetes;c__Spirochaetes	0.0000	0.0257
k__Bacteria;p__Thermotogae;c__Thermotogae	0.0000	0.0101
k__Bacteria;p__WWE1;c__WWE1_uncl	0.0000	0.0001

**Figure 4:**
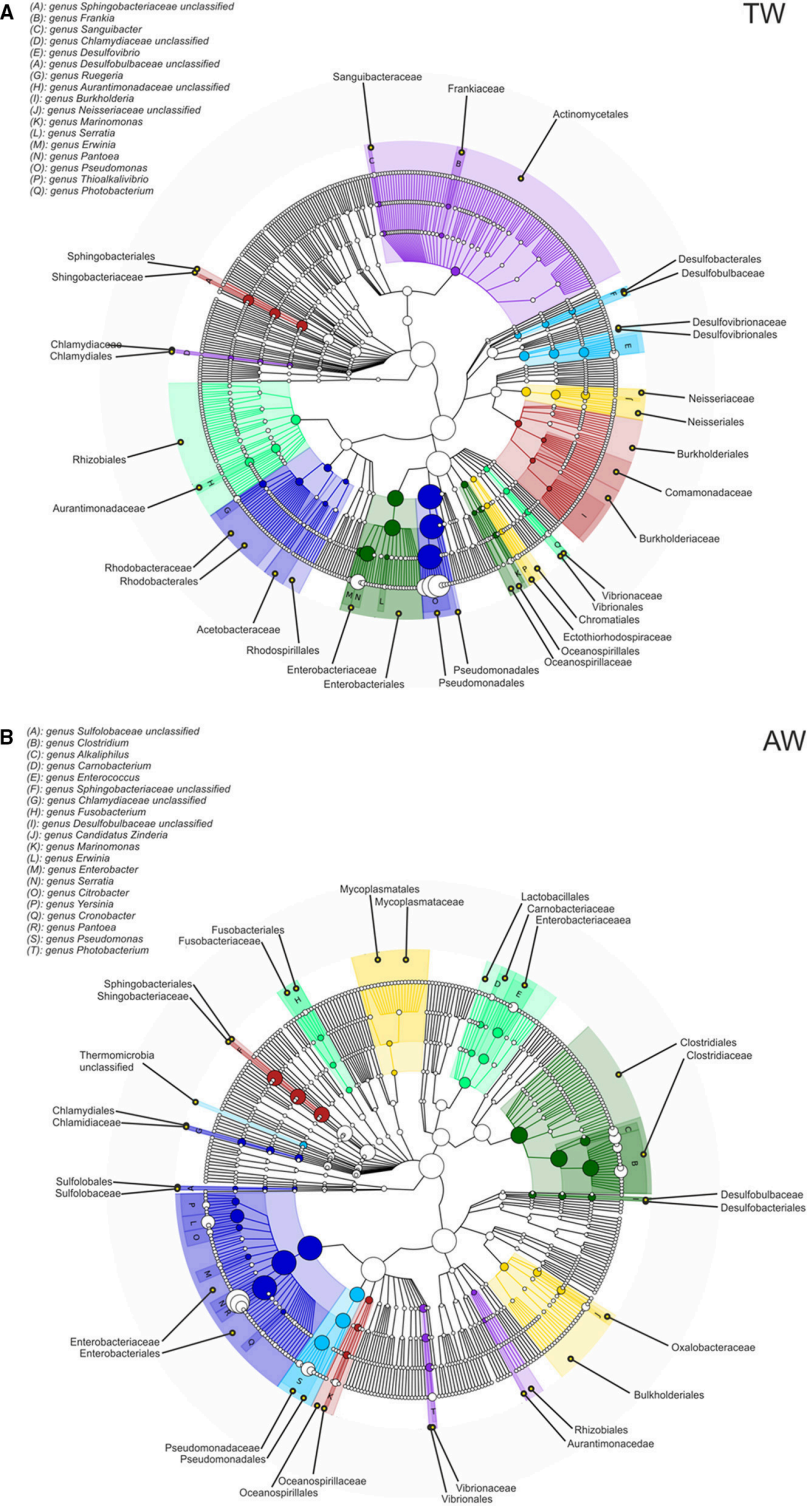
**Taxonomic cladogram reporting all clades present in the traditional (A-TW) and accelerated (B-AW) withered berry samples**. Circle size is proportional to the log of the average abundance; colors represent microbial species comprised in the same taxonomic group.

As depicted by the nodes in the cladograms of Figure 4, both samples were dominated by Pseudomonadales and Enterobacteriales, belonging to the order Gammaproteobacteria, although with different relatives abundances. Indeed, members of the family Pseudomonadaceae were present in higher levels in the TW sample, and in particular the genus *Pseudomonas* accounted for 88% of the taxa, while the high incidence of Enterobacteriaceae on the AW berries was related to a relevant abundance of the genus *Pantoea* (76%) and a moderate presence of the genus *Erwinia* (3%; Table S2). Among the minority classes, Lactobacillales were detected in the AW sample, represented mainly by the genera *Enterococcus* (0.9%) and *Carnobacterium* (0.3%; Table S2).

### Assembly of individual genomes

The assembly of reads was conducted to reconstruct the near-complete draft genomes of the bacterial species that dominate on the berries. Indeed, their abundance can be directly determined aligning the shotgun reads on the genomes and, most importantly, it is possible to infer their metabolic properties by examining their gene content (Albertsen et al., 2013).

To estimate the coverage of the scaffolds in each dataset, all the reads were aligned on the scaffolds with the Bowtie2 software: this operation revealed that the samples were effectively characterized by the presence of a relatively small amount of genomes and the populations were differentially represented in the two samples, as they had highly different coverage of scaffolds in each dataset. The tetranucleotide identity was calculated to further refine the multiple species that could be included in the same coverage-defined subset, and the conserved essential single-copy marker genes were identified (Albertsen et al., 2013).

**Figure 5:**
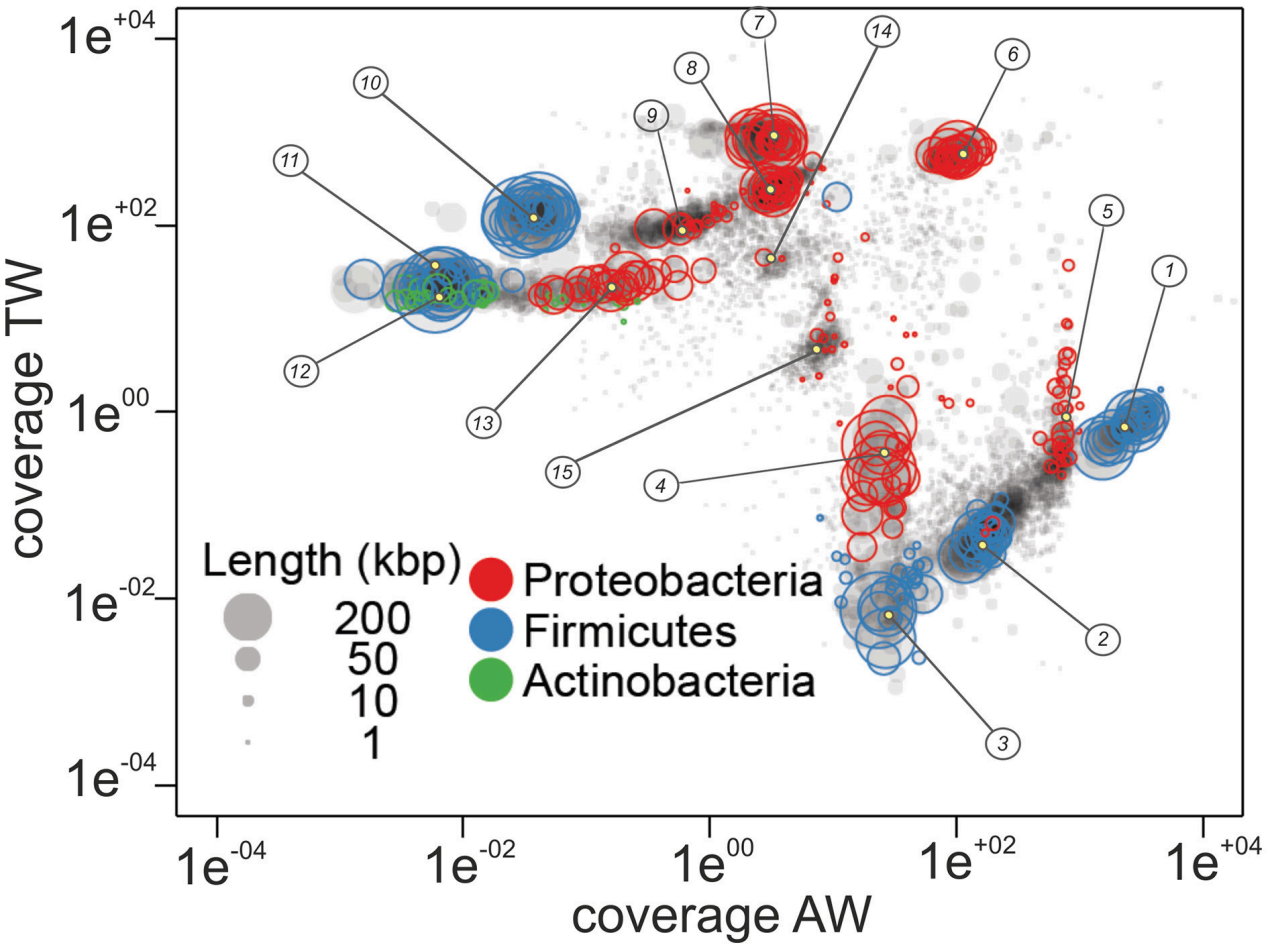
**Sequence composition-independent binning using metagenome coverage of the traditional (TW) and accelerated (AW) withered berry samples**. The nodes represent scaffolds and the color of circles around nodes indicates the phylum. The circle size is proportional to the scaffold bp content. Numbered circles represent potential genome bins.

The two coverage values were plotted against each other for all the scaffolds to achieve the binning of scaffolds into population genomes (Figure 5). Clusters of scaffolds showed in Figure 5 represented putative population bins, which captured 68% of the entire assembly, 80% and 87% of all the sequenced reads in the AW and TW samples, respectively. In total, 15 population bins were identified representing three bacterial *phyla* (Proteobacteria, Firmicutes, and Actinobacteria) with extremely different abundances in the two samples: population bins 1–5 were more abundant in the sample related to the AW grape condition, while population bins 6–14 were more represented in the dataset deriving from the TW condition. Only the population bin 15 showed similar abundance in both the metagenomic datasets.

The number of conserved essential genes determined in the first stages of the binning process indicated a low level of completeness of the population bins 14 and 15 (lower than 20%). For this reason and for their small genome size they were not included in further analysis.

Genes annotated in genomes 1–13 were also used for BLASTn analyses against *nr* database reference genomes and sequence similarity values of 95, 85, and 75% were used for species, genus and phylum level taxonomic annotation, respectively (Nielsen et al., 2014). As some genomes could not be assigned to a genus or a species by DNA similarity, they were taxonomically annotated by similarity to the Uniprot database (BLASTp, best hit, *E* < 0.001). Considering these thresholds, it was possible to ascribe genomes 4, 5, and 8 at the species level, i.e., *Erwinia billingiae, Pantoea vagans*, and *Pseudomonas syringae*, respectively; genomes 1, 2, 6, 7, 9–11, and 13 at the genus level (1, 2, *Clostridium* spp.; 6, *Pantoea* spp.; 7, 9, 13, *Pseudomonas* spp.; 10, 11, *Paenibacillus* spp.); genomes 3 and 12 at the order level, i.e., Lactobacillales and Actinomycetales, respectively (Table 3).

**Table 3:** **Assembly information of the 13 extracted genome bins**.

**Metagenomic dataset**	**Figure ID^a^**	**Phylogenetic affiliation**	**No. scaffolds**	**Total length (bp)**	**GC (%)**	**No. essential genes**	**Completeness (%)^b^**	**Coverage^b^**	**BlastP (%)^c^**	**BlastN (%)^c^**
AW	1	*Clostridium* sp. UNIVR01 strain	112	4,456,330	30.9	105/105	100	2521.4	81.5	66.4
AW	2	*Clostridium* sp. UNIVR02 strain	103	5,268,949	29.1	105/105	100	2521.4	89.3	86.5
AW	3	Lactobacillales sp. UNIVR03 strain	270	3,864,387	42.3	72/105	69	645,474.2	83.3	62.1
AW	4	*Erwinia billingiae* UNIVR04 strain	173	4,663,534	54.7	80/105	76	137.2	99.4	98.1
AW	5	*Pantoea vagans* UNIVR05 strain	189	4,663,534	55	40/105	38	1448.1	99.2	97.2
TW	6	*Pantoea* sp. UNIVR06 strain	172	4,234,112	55.4	89/105	85	8.6	93.9	87.8
TW	7	*Pseudomonas* sp. UNIVR07 strain	233	8,444,622	60.6	66/105	63	630.3	93.1	88.1
TW	8	*Pseudomonas syringae* UNIVR08 strain	177	4,540,557	59.2	68/105	65	97	98.6	97.1
TW	9	*Pseudomonas* sp. UNIVR09 strain	224	4,145,951	59.2	14/105	13	274.4	94.1	90.05
TW	10	*Paenibacillus* sp. UNIVR10 strain	57	5,684,618	40.9	68/105	65	5042.8	81.7	86.5
TW	11	*Paenibacillus* sp. UNIVR11 strain	70	6,879,406	45.9	104/105	99	5042.8	84	63.8
TW	12	Actinomycetales sp. UNIVR12 strain	246	3,863,727	63.9	80/105	76	4390	83.2	75.7
TW	13	*Pseudomonas* sp. UNIVR13 strain	96	3,898,344	60.4	75/105	71	207.9	92.4	89.1

The number of the essential genes was estimated using 105 HMM models protein coding essential single copy genes conserved in 95% of all bacteria (Dupont et al., 2012).

a Figure ID correspond to the number in Figure 5.

b Genome bins completeness and coverage were calculated as described in the Section Materials and Methods.

c BlastP and BlastN similarity values based on essential genes.

The identified population bins represented a wide bacterial diversity, including species belonging to several families (Clostridiaceae, Enterobacteriaceae, Enterococcaceae, Microbacteriaceae, Paenibacillaceae, Pseudomonadaceae) which is also showed in the phylogenetic tree based on >400 proteins optimized from among 3,737 genomes (Segata et al., 2013; Figure S2).

Focusing on OTU classification of population bins, and their coverage in the two metagenomic datasets, the TW condition particularly promoted *Paenibacillus* spp., members of order Actinomycetales (more than 4,000 and ~3,700 fold more abundant than the AW conditions, respectively), and *Pseudomonas* spp., while the AW condition favored Lactobacillales, *Clostridium* spp., and *Pantoea* spp. (~4,000, ~2,400, 740 fold more abundant than the TW condition, respectively; Table S3). The functional properties of the genome bins was investigated and the abundance of the COG categories in each genomes was reported in Table S4. In particular, the E and V categories mainly characterized *Clostridium* spp. and Lactobacillales for the AW sample and *Paenibacillus* spp. for the TW sample.

## Discussion

A number of studies have chiefly demonstrated that the microbial communities on grape surfaces play an important role in grape quality, yield, and in winemaking, contributing also to a regional terroir (Barata et al., 2012; Bokulich et al., 2014; Capozzi et al., 2015; Zarraonaindia et al., 2015). Indeed, the first population encountered by grape must prior to the fermentation can crucially affect the metabolic profile of wine and its quality, even when commercial starters are used (Bokulich et al., 2013). However, the composition of microbiota associated with withered berries prior to the onset of fermentation has not yet been investigated in details. WMS approach was used in this study, for the first time, to profile the microbial consortia populating the surface of cv. Corvina sound berries at the end of 2–3 months post-harvest withering process, and their diversity according to the different drying conditions of the *fruttaio*. We used only healthy undamaged berries both to avoid contamination by grapevine DNA and, especially, to have a picture of the microbial contaminants of withered berries without any “enrichment” due to the leakage of juice. It is crucial for Amarone winemakers to use healthy grape for the withering in order to avoid unwanted mold development. Indeed, the protocol applied to collect the microbiota of withered berry surface allowed to almost completely eliminate the presence of grapevine genetic material, maximizing the number of sequences useful for the analyses.

In addition, the WMS provided access to the functional gene composition of microbial communities, sequencing the majority of available genomes, and to the microbial phylogenetic profile among rare and abundant prokaryotic and eukaryotic sequence groups, avoiding the limitations associated to the PCR biases of the amplicon sequencing approach.

Considering functional analysis, only five major gene categories (E, G, K, U, and V) appeared differently abundant between the TW and AW berry communities: genes associated with defense mechanisms, and aminoacid metabolism and transport being relatively more abundant in the TW sample; transcription, carbohydrate metabolism, and transport, intracellular trafficking, secretion and vesicular transport in the AW sample. Such scarce variance in the overall composition of the COG functional classes suggests a high redundancy in the functional profiles characterizing the microbial communities in these two withering conditions, that may be more comparable than assumed from their taxonomic diversity and composition. Indeed, distinct taxa can share specific functional attributes and have similar physiologies and environmental tolerances (Fierer et al., 2012).

While limited differences in the distribution of genes and functional diversity were found in relation to the distinct conditions of withering process, greater evident differences were observed in the abundance of specific microbial groups in the two berry samples. In particular, this study revealed that the relative abundance of prokaryotic populations was considerably higher than that of eukaryotic populations in the berry microbiota.

Regarding the eukaryotic communities, the two most abundant genera in both samples were *Aspergillus* and *Penicillium*. The presence of such saprophytic filamentous fungi is likely due to their ability to rapidly colonize different grapevine tissues, including grape surfaces (Bokulich et al., 2013; Rousseaux et al., 2014). Indeed, spores of these molds are spread all over grapevine tissues and germinate when temperature and humidity are appropriate, especially when berries are injured (Barata et al., 2012). In addition, the withering in *fruttaio* easily exposes the grape to post-harvest contamination by airborne fungal spores. The substantial abundance of *Aspergillus* and *Penicillium* on withered grapes of cv. Garganega and Corvina has been previously reported using culture-dependent methods (Lorenzini et al., 2013). However, their incidence can be extremely variable, depending on seasonal conditions and withering techniques. The major presence of such fungal genera on the surfaces of berry collected from the TW sample was foreseeable, since it could be related to the longer permanence in *fruttaio* and the different storage conditions respect to the AW process.

An important genus associated with withered grape, i.e., *Botryotinia*, that includes *Botryotinia fuckeliana* (anamorph: *Botrytis cinerea*), was found at levels < 10% of the Ascomycota fraction in both conditions. The great interest for this mold is due to its ambivalent nature: it is widely recognized as the causative agent of gray mold, that causes severe damage on grape, but also as “noble rot,” used for processing some speciality wines (Fournier et al., 2013). Noble rot symptoms seem to depend essentially on microclimatic conditions (Blanco-Ulate et al., 2015), which has applicable consequences for the production of traditional botrytized sweet wines, like Souternes, Tokaji Aszù, and Auslese (Magyar, 2011). The effects of noble rot on the overall quality of passito red wine, like Amarone, have been less investigated. Nevertheless, it was shown that whitered Garganega and Corvina berries naturally or artificially infected with *B*. *cinerea* produced wines with distinctive organoleptic properties (Tosi et al., 2012; Azzolini et al., 2013; Lorenzini et al., 2013). In addition, Lorenzini et al. (2013) demonstrated that some *Penicillum* species are able to grow under withering conditions and have a synergic effect with *B. cinerea* on berry dehydration in simultaneous infection trials. Of particular note is also the observed antagonistic activity of *B. cinerea* vs. ocratoxin A (OTA)-producing *Aspergillus* and its capability to degrade this mycotoxin that may explain the low levels of OTA in noble wines (Valero et al., 2008). In this study, the environmental conditioning of the *fruttaio* was settled up to reproduce the traditional characteristics provided by natural drying, but assuring better control using a Natural Super Assisted Drying system, named NASA, that bring the attic to the more suitable withering conditions (Paronetto and Dellaglio, 2011). Under these conditions, the noble rot infection has been reported to occur in a limited part of the berries (Tosi et al., 2012). The detection of the genus *Botryotinia* in the two berry samples provides a steady indication of its involvement in withering process.

The other main classes characterizing the eukaryotic communities were represented by Sordariomycetes and Dothideomycetes which contain putative plant pathogens, such as *Gibberella, Pyrenophora*, and *Phaeosphaeria* spp. (Penton et al., 2014). However, as noted by Taylor et al. (2014), the presence of DNA from these genera does not necessarily mean that the grapes or plants had an infection, but provide an indication of potential disease load.

Microorganisms belonging to the order Saccharomycetales, that includes *Saccharomyces* and non-*Saccharomyces* yeasts of primary relevance for the wine fermentation process, represented a minority of the Ascomycota fraction of the samples. However, the identification of the genus *Saccharomyces* on the withered berry surfaces is interesting, especially because no enrichment steps were applied to collect the microbial community. Indeed, the detection of *Saccharomyces* species, especially *S. cerevisiae*, from sound berries has been reported very rarely, and only after the application of enrichment techniques (Cordero-Bueso et al., 2011; Barata et al., 2012). Although the frequency of occurrence of these yeasts increase up to 25% in heavily damaged berries, where grape juice became accessible to the yeasts through the skin lesions (Mortimer and Polsinelli, 1999), their origin are still poorly understood. In addition to grape, winery surfaces have been reported to harbor large population of *Saccharomyces*, potentially serving as vector of these yeasts in wine fermentations (Bokulich et al., 2013); however, to date no investigations have been carried out to monitor the microbial communities of the *fruttaio* environment. In addition, insects, such as bees, wasps, and *Drosophila*, as well as birds, can facilitate their dispersal on vineyard and winery environments (Francesca et al., 2012; Stefanini et al., 2012; Lam and Howell, 2015). According to Lynch and Neufeld (2015), *Saccharomyces* and other technological species, which are detected as rare viable or dormant microbial taxa in certain samples, can be defined as “conditionally rare taxa,” since their abundance increases when the environmental conditions change (i.e., during fermentation).

Regarding the prokaryotic population, we found the predominance of environmental ubiquitous microorganisms, i.e., Gammaproteobacteria (Pseudomonadales and Enterobacteriales), Clostridia, Sphingobacteria, and Bacilli, rather than bacteria usually associated to wine microbial consortia. Recent ecological studies using 16S-based high-throughput sequencing techniques detected several of these taxonomic groups on fresh grape samples of cv. Grenache and Carignan (Portillo et al., 2015), in musts of Chardonnay and Cabernet Sauvignon (Bokulich et al., 2014), and also during botrytized wine fermentations (Bokulich et al., 2012). The question of whether these bacteria are truly metabolically active in wine and capable of affecting the sensory quality has been raised (Bokulich et al., 2012) but it has not been studied in depth yet. The main source of such microorganisms is likely the grapevine phyllosphere, since members belonging to the above classes represent the usual microbiota linked to grapes, leaves, flowers, and soil of *V. vinifera* (Martins et al., 2013; Gilbert et al., 2014; Pinto et al., 2014; Rolli et al., 2015). In addition, harvest, transfer and storage of grape represent all processing stages for further contaminations (Bokulich et al., 2013), and especially the *fruttaio* habitat could be an important reservoir of environmental microbial species for withered berries.

Interestingly, the conditions of the drying process strongly influenced the relative abundance of members of the families Enterobacteriaceae and Pseudomonadaceae; indeed, the faster grape drying favored the genera *Pantoea* and *Erwinia*, while *Pseudomonas* was more abundant in the traditional drying sample. These differences revealed by shotgun reads analysis were further confirmed by the assembly and binning processes, which allowed the assignment of three genome bins to the species level: *P. vagans, E. billingiae*, and *P. syringae*. Species of *Pseudomonas* and *Erwinia* were recently found on grapevine leaves of the *V*. *vinifera* cv. Pinot gris, and were considered to represent the phyllosphere core bacterial community (Perazzolli et al., 2014). As reported in several studies, *Pseudomonas* taxa are characterized by some positive physiological features, like the capability to produce exopolysaccharides and antifungal compounds, that can contribute to the maintenance and protection of the microbial communities present on berry surfaces (Trotel-Aziz et al., 2008; Verhagen et al., 2010; Martins et al., 2012). Also the genus *Pantoea* has often been described within the grapevine microbiome and has been proposed as bacterial antagonist with biocontrol ability (Trotel-Aziz et al., 2008; Bulgari et al., 2009). In particular, the plant-associated non-pathogenic *E. billingiae* and *P. vagans* are able to compete with different plant pathogens, e.g., *Erwinia amylovora* (Kube et al., 2010; Smits et al., 2010). *P. syringae* is a common foliar bacterium that can be responsible of extensive yield losses in wine-grape production (Hall et al., 2015); however, strains of this species were also found as harmless commensals on leaf surfaces, and their capability to produce the surfactant syringomycin can improve their adaptation to phyllosphere habitat (Whipps et al., 2008). Competition for space and nutrients, production of hydrolytic enzymes, inhibition of pathogen-produced enzymes or toxins, and, in general, direct and indirect interactions between microorganisms resident on berry surface can surely affect biodiversity, favoring the survival of certain microbial species, but actually the involved factors have not yet been clearly identified.

*Lactobacillales* is the most important bacterial order in wine fermentation, being involved both in spoilage and malolactic activity (Bokulich et al., 2012). However, it was detected as a minor bacterial taxon (1.3%) of the AW sample and comprised only the genera *Enterococcus* and *Carnobacterium*. Enterococci are environmental ubiquitous bacteria which have been isolated, although not frequently, from the surface of grape berries at harvest (Renouf et al., 2005), and wine undergoing malolactic fermentation (Barata et al., 2012; Pérez-Martín et al., 2014). Capozzi et al. (2011) has proposed that the origin of these bacteria are the grapes, the winery equipment or practices. Conversely, the genus *Carnobacterium* was not usually associated with grape and the winemaking process, although it has been recently found at low level in Portuguese wines (Pinto et al., 2015), and strains of *Carnobacterium viridans* and *Carnobacterium inhibens* were isolated and identified from wine wooden vats (Fracchetti et al., 2015). The role of the genera *Enterococcus* and *Carnobacterium* in grape and wine is, until now, unknown perhaps as a consequence of their scarceness.

Most of the putative genomes were assigned to the genus level (*Clostridium, Paenibacillus, Pantoea*, and *Pseudomonas*), but not to the species level, likely due to the challenging in the assignment of the 16S rRNA gene to the correct genome. For this reason, the sequence of the 16S rRNA gene was not used as a phylogenetic marker, but the taxonomic assignment was entirely based on previously identified phylogenetic marker genes, either clade-specific or universal, and rarely subject to horizontal gene transfer (Dupont et al., 2012; Albertsen et al., 2013; Segata et al., 2013). Otherwise, these genome bins could represent new taxa for which the *Candidatus* provisional status may be proposed (Konstantinidis and Rosselló-Móra, 2015). A modification of the current binning strategy assisting the assignment of the 16S rRNA sequences to the genomes is probably needed. This can improve the reliability of the taxonomic assignment by taking advantage of the high number of 16S rRNA sequences present in public databases and extending the potential of the single-copy marker genes that, however, it was proven to be very good (Mende et al., 2013; Sunagawa et al., 2013). Despite this, it has to be considered that the binning approach can provide fundamental insights into physiological potential of the species identified, while the 16S rRNA analysis can only be used for the taxonomical analysis. The accessibility of these near-complete genomes could also provide valuable information about the nutritional requirements of these microorganisms in order to define a proper cultivation medium for their isolation. The availability of isolates could be useful for mainly two reasons: to evaluate whether the same strain is persistent over different vintages (contribution to the microbial component of the *terroir*) and to perform experiments of controlled inoculum on berries. This could give an important insight on the relationship between microbial component and grape metabolites produced during berry withering.

In conclusion, data presented here provide new insights into the complex microbial consortium of withered sound grape of cv. Corvina, indicating that the core microbiota associated with berry surfaces at the end of withering is mainly constituted by environmental rather than microorganisms relevant for wine production. However, “conditionally rare taxa,” like *Saccharomyces*, were also detected. Interestingly, withering conditions had a strong influence on the taxonomic composition and abundance of grape microbiota, but the abundance of the functional classes did not undergo a profound modification. It could be guessed that the different abiotic factors (e.g., temperature, humidity, ventilation) applied during withering have determined more subtle damaging effects in the berries of the AW batch, leading to a release of nutrients. This in turn may impact the microbiota present on the damaged berry surface, causing a higher diversity and favoring some fermentative populations. Such microorganisms could be spread or carried by wind generated by the fan to adjacent healthy berries, including those of the AW sample.

Further studies have to be performed to determine whether the modification of the microbial communities on grape surfaces withered under diverse conditions could lead to significant chemical variations of Corvina berry metabolites, thus influencing the final wine characteristics and sensory attributes. In this way, WMS could open novel perspectives in the knowledge and management of traditional processes, such as the withering process of Corvina grape, with an impact on the winemaking of important Italian wines.

## Author contributions

Conceived and designed the experiments: ES, ST, FF, GT, GF. Performed the experiments: ES, SC, AG, FF. Generated and analyzed the data: ES, SC, AG, IC, GT, GF. Wrote the paper: ES, SC, IC, ST, GF.

### Conflict of interest statement

The authors declare that the research was conducted in the absence of any commercial or financial relationships that could be construed as a potential conflict of interest.
