# Supplementary material for: Whole-Metagenome-Sequencing-Based Community Profiles of Vitis vinifera L. cv. Corvina Berries Withered in Two Post-harvest Conditions
Source: Front Microbiol. 2016 Jun 23;7:937. doi: 10.3389/fmicb.2016.00937 (PMC4917526; doi:10.3389/fmicb.2016.00937)
Supplement: Supplementary file 5 [file DataSheet1.PDF]

Supplementary material

Whole-metagenome-sequencing-based community profiles of *Vitis vinifera* L. cv. Corvina berries withered in two environmental conditions

Elisa Salvetti[†], Stefano Campanaro[†], Alex Gobbi, Ilenia Campedelli, Fabio Fracchetti, Giovanni Battista Tornielli, Sandra Torriani[*], Giovanna E. Felis

[†]These authors have contributed equally to this work

[*]**Correspondence**:
Sandra Torriani
sandra.torriani@univr.it

1 Supplementary Figures and Tables

1.1 Supplementary Figures

Figure S1. Overview of the scaffold size obtained through the assembly process and their bp content.

Figure S2. Phylogenetic tree based on >400 proteins optimized from among 3737 genomes. It includes the 15 genome bins obtained in this study, highlighting that the population bins belong to the families Clostridiaceae, Enterobacteriaceae, Enterococcaceae, Microbacteriaceae, Paenibacillaceae, Pseudomonadaceae.

1.2 Supplementary Tables

Table S1. Annotation of genes identified in the metagenomic assembly and assignment of the scaffolds to the microbial genomes. The protein-encoding genes identified with Prodigal were annotated using different procedures (see Methods for details). In columns (A-AA) are reported: (A) scaffold; (B) gene ID as “scaffold_gene number”; (C) genome bins assignment (names and description of the genome bins are reported in Table 3); (D) COG ID; (E) COG ID “short code”; (F)

e-value of BLAST results for COG; (G) gene name, (H) gene function; (I) KEGG ID; (J) link to the KEGG function; (K) EggNOG gi code; (L) EggNOG ID; (M) EggNOG gene function; (N) e-value of eggNOG blast result; (O) gi code of blast search against NCBI microbial genomes; (P) NCBI gene ID; (Q) gene function description; (R) most similar species; (S) e-value for BLAST search against NCBI microbial genomes, (T) Pfam ID; (U) e-value for BLAST search against Pfam; (V) Pfam domain; (W-AA) Pfam domain description. “NA” = not assigned.

Table S2. Relative abundance of prokaryotic associated with grape surfaces of the TW and AW process obtained through the MetaPhlAn analyses.

Table S3. Coverage of the population bins of the TW and AW samples and their ratio.

Table S4. Percentage of proteins assigned to the COG classes for each genome bin. Percentage is calculated considering the total number of genes on each near-complete genome.
