# Supplementary figures and images for: Whole-Metagenome-Sequencing-Based Community Profiles of Vitis vinifera L. cv. Corvina Berries Withered in Two Post-harvest Conditions
Source: Front Microbiol. 2016 Jun 23;7:937. doi: 10.3389/fmicb.2016.00937 (PMC4917526; doi:10.3389/fmicb.2016.00937)

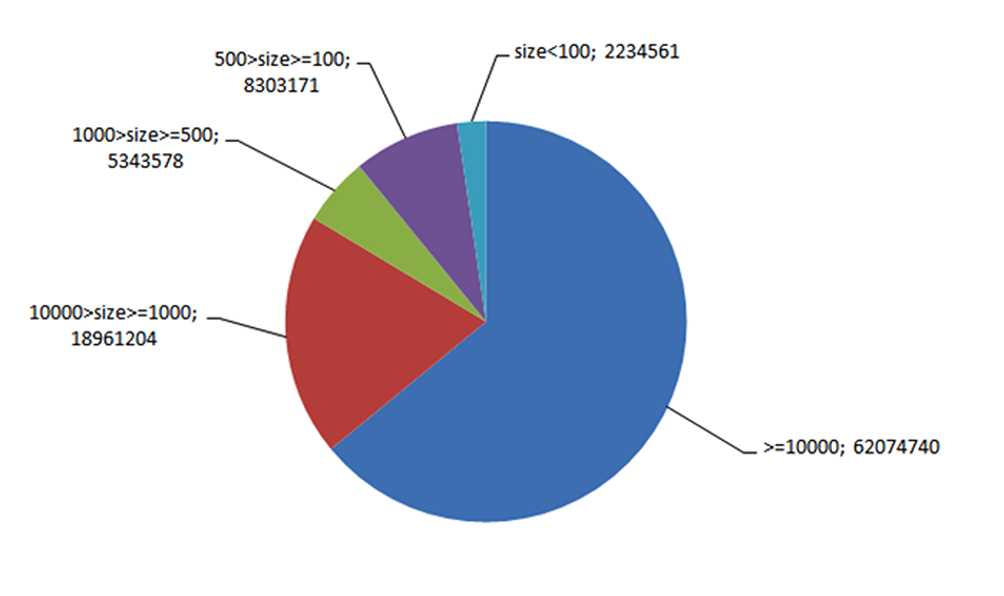

Supplement: Supplementary file 6 [file Image1.TIF]

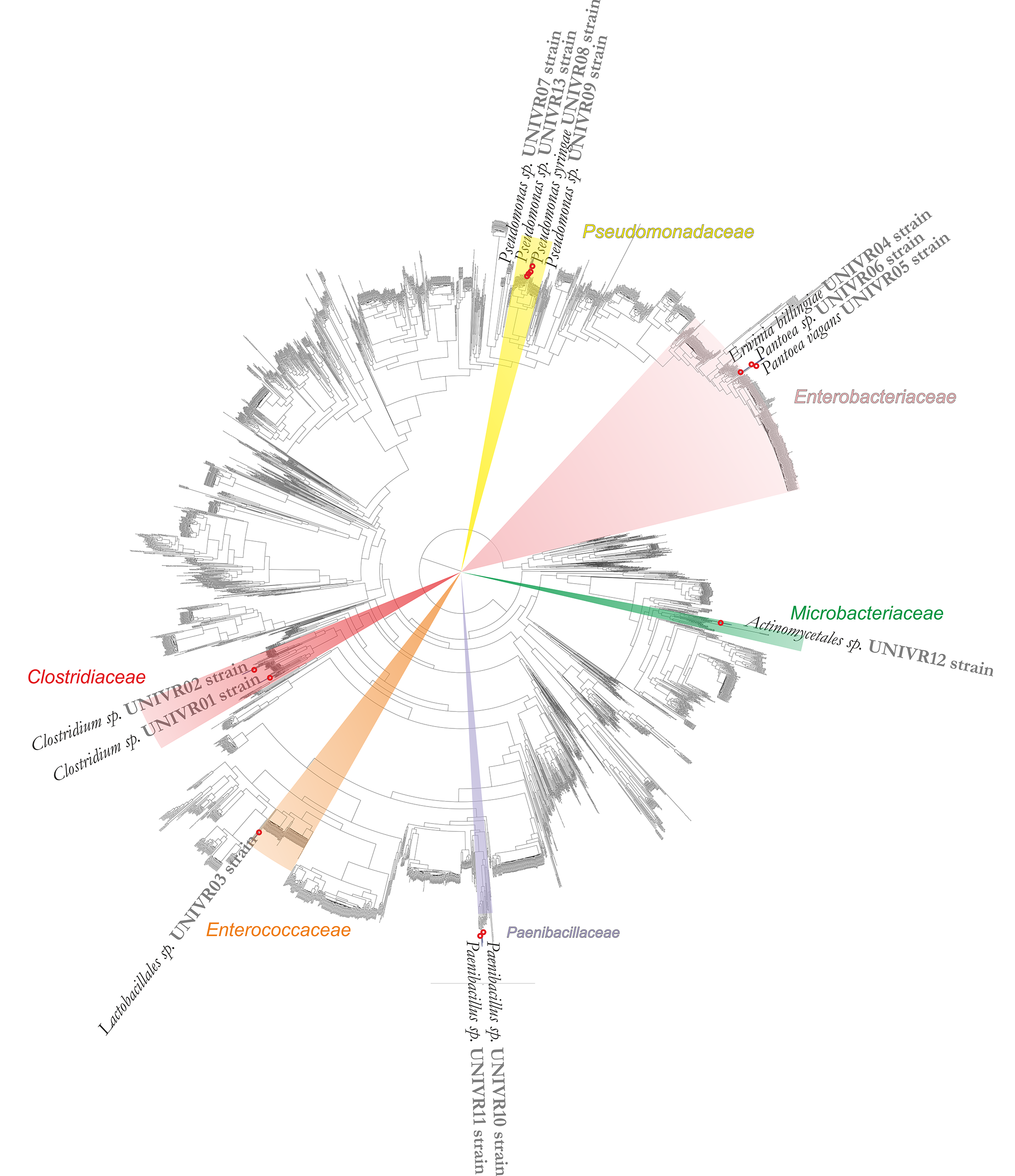

Supplement: Supplementary file 7 [file Image2.TIF]
